# OPTIMAL STAFFING MODELS TO CARE FOR FRAIL ELDERLY ADULTS IN PRIMARY CARE AND GERIATRIC PRACTICES

**DOI:** 10.1093/geroni/igz038.255

**Published:** 2019-11-08

**Authors:** David I Auerbach, Carie Michael, Douglas Levy, Peter Maramaldi, Robert Dittus, Joann Spetz, Peter Buerhaus, Karen Donelan

**Affiliations:** 1 Montana State University, Bozeman, Montana, United States; 2 Massachusetts General Hospital, Boston, Massachusetts, United States; 3 Simmons College School of Social Work, Boston, Massachusetts, United States; 4 Institute for Medicine and Public Health at Vanderbilt University, Nashville, Tennessee, United States; 5 Institute for Health Policy Studies at the University of California, San Francisco,, San Francisco, Massachusetts, United States; 6 Center for Interdisciplinary Health Workforce Studies at the College of Nursing, Montana State University, Bozeman, Montana, United States; 7 The Mongan Institute, Health Policy Research Center, Boston, Massachusetts, United States

## Abstract

As the US population ages, primary care is expected to be the health care “home” for older adults, and several initiatives are aimed at helping to transform primary care practice to care for this population. Wide variation in staffing has been observed. Meyers et al proposed ideal models of primary care staffing for a general population and for a frail elderly population (2018). We developed the 2018 Survey of Primary Care and Geriatric Clinicians to measure optimal team configuration in clinical practices caring for older adults. A majority employed NPs, MDs and PAs, with [r = -.53] between % of clinician labor of NPs and physicians). High-NP practices are more likely located in states with full scope of practice, perform well for frail elders and are less expensive. Meyers' models, with fewer physicians, more SW and CHWs, more RNs, perform better for frail elders, and are less expensive.

